# Supplementary material for: DMRforPairs: identifying Differentially Methylated Regions between unique samples using array based methylation profiles
Source: BMC Bioinformatics. 2014 May 15;15:141. doi: 10.1186/1471-2105-15-141 (PMC4046028; doi:10.1186/1471-2105-15-141)
Supplement: Additional file 2 — DMRforPairs output for the comparison of A431-MCF7 and NA17018-NA17105. Please start from the HTML files in each folder. Available via the BMC Bioinformatics website. [file 1471-2105-15-141-S2.zip › 1394847754114233_MOESM2_ESM/A431_MCF7/figures/37445.pdf]

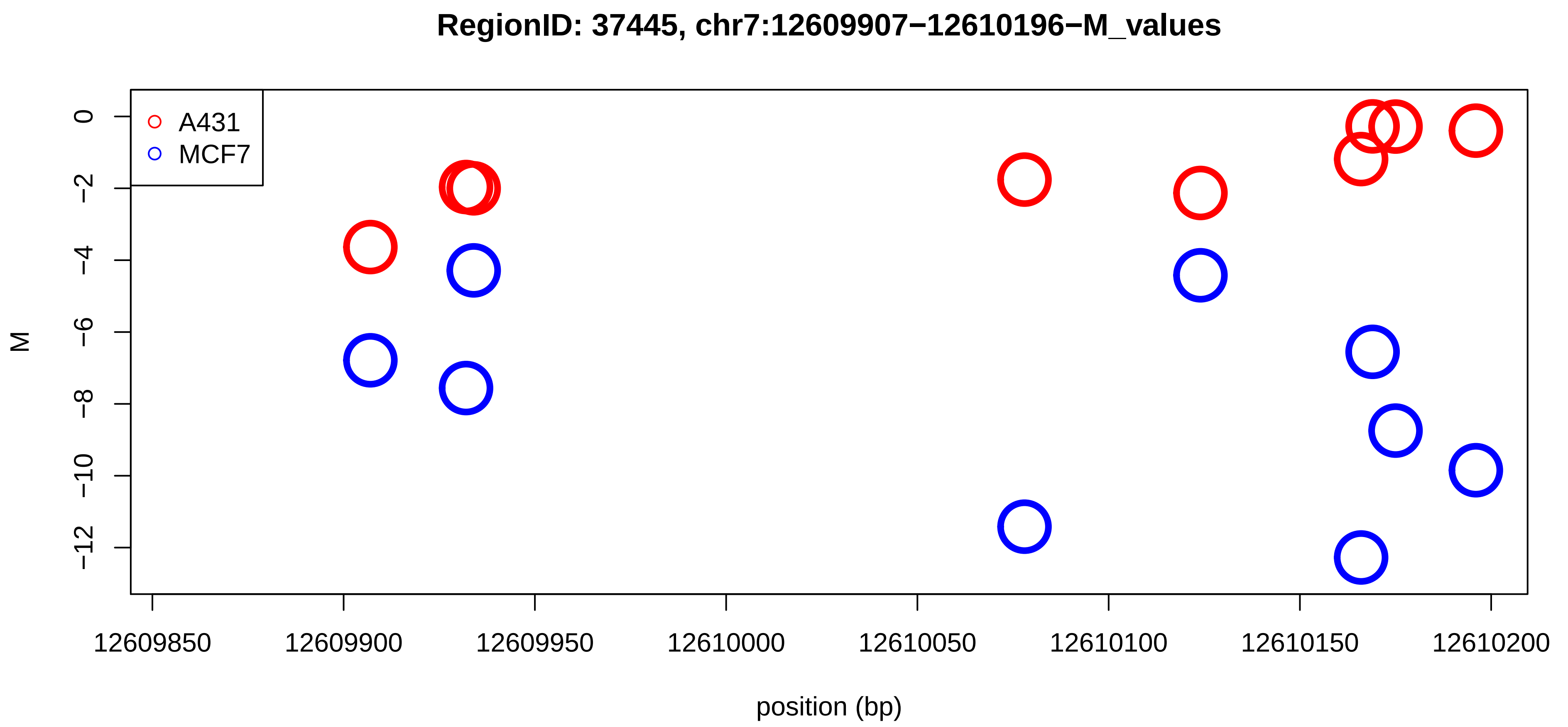
RegionID: 37445, chr7:12609907−12610196−M_values
A431
MCF7
M
0
−2
−4
−6
−8
−10
−12
12609850
12609900
12609950
12610000
12610050
12610100
12610150
12610200
position (bp)

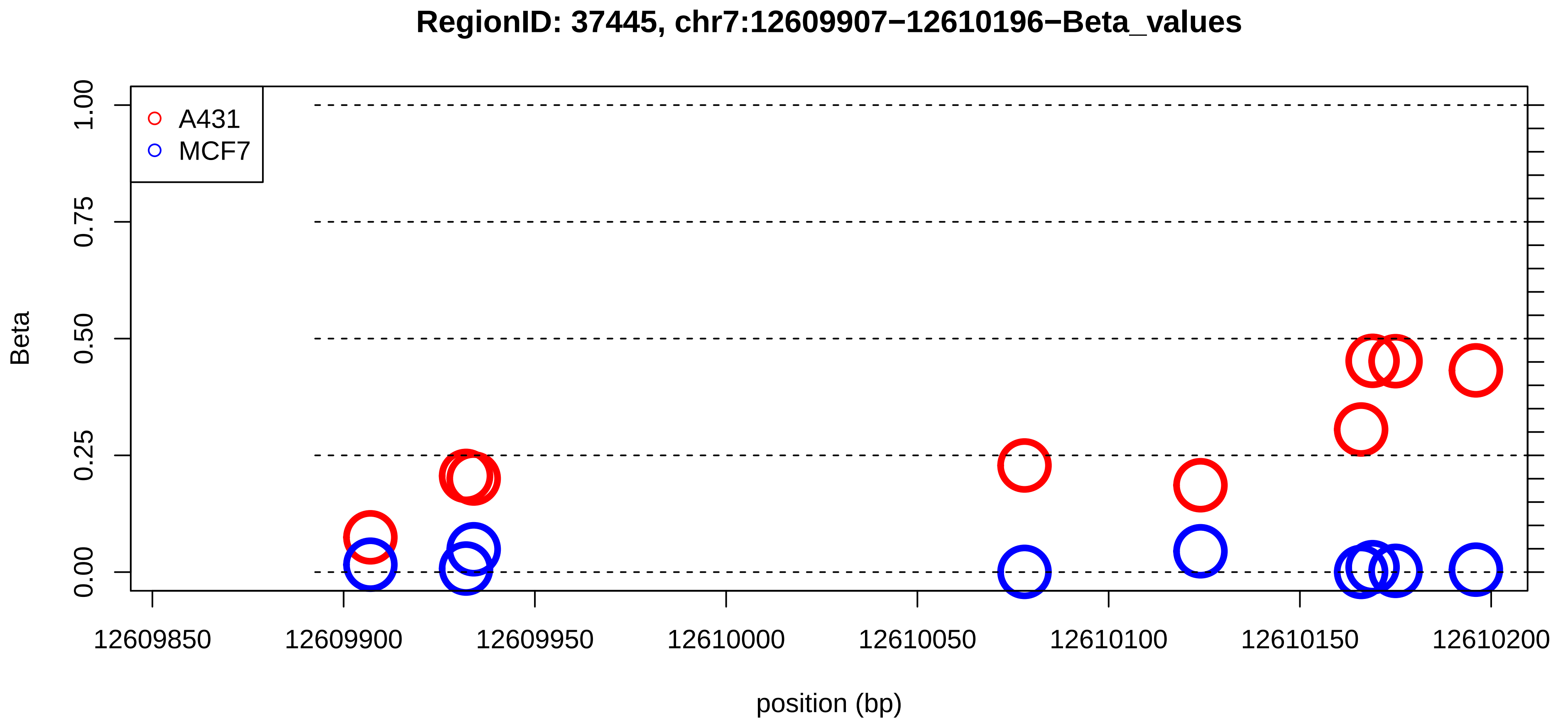
RegionID: 37445, chr7:12609907−12610196−Beta_values
A431
MCF7
Beta
0.00
0.25
0.50
0.75
1.00
12609850
12609900
12609950
12610000
12610050
12610100
12610150
12610200
position (bp)

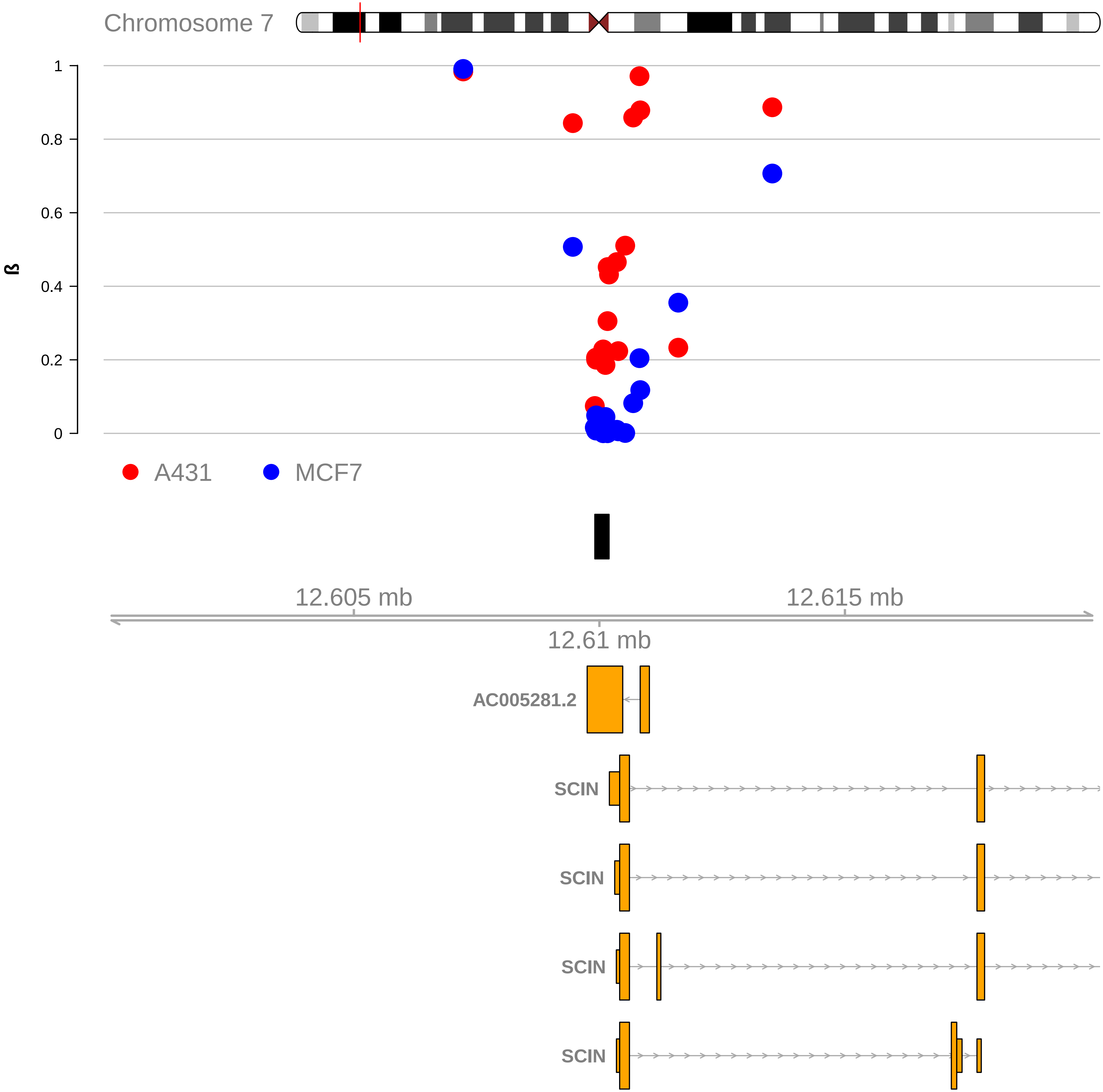
Chromosome 7
1
0.8
0.6
0.4
0.2
0
ß
A431
MCF7
12.605 mb
12.615 mb
12.61 mb
AC005281.2
SCIN
SCIN
SCIN
SCIN
